# Developing Leaders: Implementation of a Peer Advising Program for a Public Health Sciences Undergraduate Program

**DOI:** 10.3389/fpubh.2014.00288

**Published:** 2015-01-05

**Authors:** Megan Griffin, Gloria T. DiFulvio, Daniel Shea Gerber

**Affiliations:** ^1^Department of Public Health, University of Massachusetts Amherst, Amherst, MA, USA; ^2^School of Public Health and Health Sciences, University of Massachusetts Amherst, Amherst, MA, USA

**Keywords:** advising, peer mentor, undergraduate, student success, benefits, program

## Abstract

Peer advising is an integral part of our undergraduate advising system in the Public Health Sciences major at the University of Massachusetts Amherst. The program was developed in 2009 to address the advising needs of a rapidly growing major that went from 25 to over 530 majors between 2007 and 2014. Each year, 9–12 top performing upper-level students are chosen through an intensive application process. A major goal of the program is to provide curriculum and career guidance to students in the major and empower students in their academic and professional pursuits. The year-long program involves several components, including: staffing the drop-in advising center, attending training seminars, developing and presenting workshops for students, meeting prospective students and families, evaluating ways to improve the program, and collaborating on self-directed projects. The peer advisors (PAs) also provide program staff insight into the needs and perspectives of students in the major. In turn, PAs gain valuable leadership and communication skills, and learn strategies for improving student success. The Peer Advising Program builds community and fosters personal and professional development for the PAs. In this paper, we will discuss the undergraduate peer advising model, the benefits and challenges of the program, and lessons learned. Several methods were used to understand the perceived benefits and challenges of the program and experiences of students who utilized the Peer Advising Center. The data for this evaluation were drawn from three sources: (1) archival records from the Peer Advising Center; (2) feedback from PAs who completed the year-long internship; and (3) a survey of students who utilized the Peer Advising Center. Results of this preliminary evaluation indicate that PAs gain valuable skills that they can carry into their professional world. The program is also a way to engage students in building community within the major.

## Introduction

For most of its history, public health education focused on graduate studies. Over the last 10 years there has been a surge across the country of undergraduate public health education programs in response to both student interest and a need to develop a strong public health workforce. At the University of Massachusetts Amherst (UMass), School of Public Health and Health Sciences (SPHHS), a new undergraduate major in Public Health Sciences (PHS) was approved in 2007 to meet this growing demand. Starting with 25 students, the major has increased to over 530 students in 7 years and continues to be one of the fastest growing majors on campus. Meeting the advising needs of these 530 undergraduate students is a significant challenge, especially for a program that historically focused on advising graduate students. Additionally, students must navigate a complex set of major and university requirements and career preparation alternatives. To meet these needs, the Department of Public Health at the UMass instituted a peer advising program to help meet the growing need for student advising, provide peer mentorship for students, and provide students with leadership development opportunities in public health.

Several authors report positive outcomes for both the mentor and mentee from peer advising and other peer mentoring programs (1–5). Positive outcomes include increased retention (6) and overall general satisfaction with their academic program (7–10), as well as positive impact on the peer advisors (PA) (11–13). While such research exists, the value of such programs in the discipline of public health has not been explored.

The purpose of this paper is to discuss the Department of Public Health undergraduate peer advising model, including the benefits and challenges of the program, and lessons learned. Our primary guiding question was the following: What are the perceived benefits and challenges of the program from the perspective of the PA? A secondary question focuses on the benefits of the PA program for advisees based on preliminary findings from a survey of students who utilized the Peer Advising Center.

## Background and Rationale

### University of Massachusetts Public Health Sciences and peer advising model

The PHS Undergraduate Program is housed in the Department of Public Health within the CEPH accredited SPHHS. The program has a part-time Faculty Director and one full-time Undergraduate Advisor. The Undergraduate Program Director is responsible for the overall curriculum, faculty development, study abroad and internship opportunities, and meeting with students that have more complex issues. The Program Advisor is responsible for the Peer Advising Program and advising undergraduate students. For the most part, juniors and seniors are strongly encouraged to make an appointment with the Undergraduate Program Advisor or the Undergraduate Program Director to make sure they are on track to graduate. Students may also meet with faculty to explore future career opportunities and graduate school. In addition to staff and faculty advising, peer advising is an essential part of our undergraduate advising system in the PHS major.

Two tracks are available within the PHS major: social science and science. Both tracks prepare students for entry-level public health positions and graduate school. The required public health courses in each track are designed to introduce students to five public health core competencies: community health education, health policy and management, environmental health sciences, epidemiology, and biostatistics. In addition, students in both tracks must complete a collateral requirement of 18 credits from courses of their choice that are related to the study of public health. Examples of proposed courses of study for the collateral field include public policy, sociology, biology, and psychology. Engaging students to think critically and discover his/her own passions is a key element of the program. In this process of self-discovery, students are encouraged to ask questions about how they foresee themselves making an impact in public health.

Public health is an interdisciplinary field, bringing together aspects of science, medicine, economics, sociology, politics, and social justice. Given the interdisciplinary nature of the major and diversity of the public health field, it is essential that students are provided regular access to an advisor who can provide guidance on the many paths that a student can take. It is the program’s goal that each student is aware and encouraged to take advantage of opportunities outside of the classroom to further their learning and prepare them for the public health workforce. Furthermore, being at a University with over 20,000 students, the advisors can help advisees navigate the large institution. An advisor serves as a resource to help direct students toward these opportunities.

### Public Health Sciences’ peer advising model

The UMass Peer Advising Program was initiated in 2009 to address the advising needs of a rapidly growing major. A major goal of the program is to provide curriculum and career guidance to students in the major, build community among students within the major, and empower students in their academic and professional pursuits. The year-long program is delivered and supervised by the Undergraduate Program Advisor. Course material was developed in consultation with the Undergraduate Program Director who is a faculty member and oversees the Undergraduate Program Advisor including course delivery. The course involves several components, including: staffing the drop-in advising center, attending training seminars, developing and presenting workshops and events for students, meeting prospective students and families, evaluating ways to improve the program, and collaborating on self-directed projects. The PAs also serve as advisors to the administrators of the program, providing insight into the needs and perspectives of students in the major. In turn, the model is designed to provide PAs with valuable leadership, communication skills, and strategies for improving their own success as well as the success of other students within the major.

### Recruitment and training

Each year, 9–12 upper-level students are chosen through an intensive application and interview process. In addition, to having a passion for public health, a desire to help other students, and excellent interpersonal and listening skills, PAs must meet specific eligibility requirements to apply for the position (Table 1). Selected prospective PAs must complete an application and meet with the Undergraduate Program Advisor and several PAs for an interview.

**Table 1:** **Eligibility for peer advising**.

1. Be a rising junior or senior
2. Have completed at least one full year as a public health major
3. Commit to completing a second semester of peer advising after the fall seminar
4. Have and maintain a cumulative GPA of 3.2 while serving as a PA

The PAs are hired, trained, and supervised by the PHS Undergraduate Program Advisor. Once a PA is accepted in the spring, he/she must complete the Family Education Rights and Privacy Act (FERPA) training. FERPA is the main law that protects the confidentiality of students’ records in an academic setting. During the summer, the PAs receive a manual including information about the PHS major, PHS careers, University and department resources and policies, advising rules, and opportunities within and outside the major. The students are expected to review the material and take an exam on the material during the first week of school. Additionally, they must attend an all-day training session during the first week of school and participate in community-building activities.

Two experienced PAs serve as mentors to the new PAs and oversee various responsibilities and public health events. These two Head PAs work closely with the Undergraduate Program Advisor to coordinate events, identify needs for projects, provide feedback about the new PAs, and facilitate seminars. Each new PA shadows a Head PA during the spring semester (after they are selected) and during the first week of school.

There are two primary avenues by which the PAs receive training. First, all PAs must attend an all-day training at the beginning of the fall semester. Second, all PAs attend a weekly 2.5-h seminar throughout the fall and a weekly 2-h seminar in the spring. The objectives of the seminar are diverse, providing PAs information on various components of the public health field and major, academic policies, diversity in higher education, advising techniques, communication skills, group dynamics, event planning, and community-building.

Objectives of the Peer Advising Program state that PAs will be able to:

- Define Public Health and identify the five domains of public health: Community Health, Policy and Management, Biostatistics, Epidemiology, and Environmental Health.
- Describe the highly interdisciplinary nature of the field.
- Articulate major requirements, career choices, and public health opportunities outside of the classroom.
- Help students identify courses and additional opportunities that are linked with their interests.
- Articulate the impact of social determinants on college students’ academic success.
- Develop and practice appropriate strategies in working with culturally diverse populations.
- Examine and articulate one’s own identity, values, mental models, and biases.
- Examine and articulate how mental models affect advising interactions.
- Demonstrate and integrate key concepts of coaching and motivational interviewing in peer advising.
- Identify traits and skills of successful leaders.
- Identify different modes of successful communication.
- Identify strategies to improve the PHS major.
- Identify strategies to build community among the major.

### Responsibilities and assessment

Peer advisors receive three internship credits in the fall and two in the spring. The Head PAs receive an additional credit each semester. The PAs are assessed on the completion of their responsibilities, including:

- Attending weekly seminars and assigned office hours.
- Conducting information sessions for majors and prospective majors.
- Visiting PH classes to introduce themselves.
- Attending open house to meet prospective students and families.
- Attending PHS club.
- Volunteering at SPHHS events.
- Completing test and quizzes.
- Completing weekly reflections and responses on their advising experience.
- Completing departmental service project.
- Assisting Head PAs on different projects.
- Writing a short article about public health for the PHS weekly newsletter.
- Completing a final reflection paper.

The goal is to have the peer advising drop-in center open 30–40 h per week. Each PA is expected to staff the center 3 h per week. The PAs are trained to provide PHS majors academic advising and guidance to meet department and University requirements. Before each advising session, advisees have to sign a form giving permission to the PAs to review their academic requirement report and discuss courses. Advisees are allowed to decline permission for the PA to view their academic information if they only want to ask questions. After each advising session, the PA must complete an advising log stating whom they spoke to (name of student, student ID number, year, major, and track), the reason for the visit, advice that was provided, actions taken, and referrals. The logs are reviewed on a weekly basis to ensure the correct information was provided. PAs are required to write a weekly online post about their experience in the Peer Advising Center. The posts provide a place for students to discuss and resolve issues, create cohesiveness with shared experiences, and increase feeling of support.

### Role of a PA in one-to-one advising sessions

In meeting with advisees, PAs provide guidance in the following areas:

- Understanding the field of public health and career options.
- Understanding how the major requirements help create a solid foundation for entry into the field or graduate school.
- Applying to the major.
- Course selection and/or sequencing, including appropriate courses for the collateral field.
- How to use and read academic requirements report.
- Registration procedures, add/drop deadlines, and withdrawing from courses before and after the mid-semester date.
- Obtaining and filling out collateral field and course exception forms.
- Internship, study abroad, volunteering, and Five College opportunities.
- Helping students figure out if they are completing all the necessary program and University requirements for graduation.
- Resources available to students.

Each PA must contribute to the Department, PHS student body, community, and/or SPHHS through a departmental service project. PAs have the option to collaborate with other PAs or create their own project/event. A proposal must be submitted and approved before the start of the project. Due to the success, many of the projects are repeated the following year (Table 2). Another component of the PA program is to engage in the community through the PHS club. The PAs are required to attend at least three PHS Club events and/or meetings each semester.

**Table 2:** **Peer advisor departmental service projects**.

1. Created and managed social media pages on Twitter and Facebook to communicate advising information, upcoming events, and job/internship opportunities
2. Created and led an undergraduate PHS student advisory committee to provide feedback to the faculty and staff
3. Created a networking and orientation workshop for new majors
4. Organized a PHS professor panel for students to meet their faculty and learn about their research
5. Re-formatted and edited advising forms
6. Created workshops on opportunities in the major, such as internships, studying abroad, and strategies to get involved in public health and the community
7. Created workshops on various PH issues, such as food insecurity, healthy relationships, stress reduction, and violence prevention
8. Organized events for National Public Health Week
9. Organized volunteers to visit local nursing homes and a rehabilitation center

## Methods

Several methods were used to understand the perceived benefits and challenges of the program and experiences of students who utilized the Peer Advising Center. The data for this evaluation were drawn from three sources:

(1) Archival records from the Peer Advising Center.
(2) Reflection papers from one cohort of PAs who completed the year-long internship.
(3) A survey of students who utilized the Peer Advising Center.

### Archival records

The PAs completed a record of each visit. The total number of student visits during the 2013–2014 academic year and the reason for the visit was summarized.

### Peer advisors’ reflection paper

Reflection papers from nine of the PAs (female = seven and male = two) were analyzed. Each of the PAs were required to submit a 5–10-page paper at the end of both fall and spring semesters reflecting on their experience as a PA. They were asked to reflect upon their experiences broadly, including skills learned and suggestions for departmental, advising, and training improvements (Table 3). A codebook was developed and modified as analyses progressed to reflect emerging themes or to merge thematically equivalent codes. On completion of coding, patterns in the data were identified. Recurrent themes were summarized into several broad categories. The reflection papers were also reviewed for key quotes that highlighted their experiences in the internship.

**Table 3:** **Questions PAs were requested to address in their reflection papers during fall and spring semesters 2013–2014**.

1. Describe your experience as a peer advisor
a. What was a highlight of being a peer advisor? How did your work engage and enlighten you?
b. Comment on what did and did not work, barriers, and successes
2. What new public health knowledge and skills did you acquire during your internship experience?
3. What were some highlights of the training day before the semester started? Do you have any specific suggestions for next year?
4. What are at least three suggestions to improve the course and better prepare you for being a Peer Advisor?
a. What additional classroom knowledge might have been useful?
b. Do you have any specific topics that you would like to include or exclude? Please explain why
5. Provide a critique of one of our guest speakers from this past year. Describe what you thought was most helpful, what could be changed, or be removed
6. Provide feedback on the supervision/assistance you received
a. What type of assistance was the most helpful to you?
b. Was the supervision/assistance adequate?
c. How would you like the supervision to change in the future?
d. How did you like having head PAs?
7. Comment on your participation in and out of class. What was a project that you worked on? What are you most proud of accomplishing?
8. How do you think that you can improve as a peer advisor? What are some steps that you could take to achieve your goal?
9. In what ways have you grown from your experience as a peer advisor? What specific skills or knowledge have you acquired that you plan to apply to your future career, relationships, and/or lifestyle?
10. Include any additional suggestions or comments about the peer advising team and class

### Utilization of the Peer Advising Center

In April 2014, an electronic survey was sent to 507 undergraduate public health students for input into their experiences as a Public Health major. The survey included demographics of the participants’ class year and major track. Two questions from this survey focused on their experience of using the Peer Advising Center. The first question was “How often do you seek Public Health advising with peer advisors?” Participants could select “never, once a semester, two-to-three times per semester, over four times per semester.” The second question was “Based on your last advising session with a PA, how would you rate your experience on a scale 1–5 with 1 = not good and 5 = excellent?”

## Results

### Archival records

In the 2013–2014 academic year, the PAs had a total of 573 one-to-one meetings with students in the Peer Advising Center. Of the 573 visits, sophomores visited the office most frequently (*n* = 195), followed by juniors (*n* = 130), seniors (*n* = 86), first years (*n* = 86), and unclassified (*n* = 76). The three most common reasons that students visited the Peer Advising Center were questions about course selection, applying to the major, and collateral field.

### Peer advisors’ reflection paper

#### Perceived benefits of the Peer Advising Program

Peer advisors identified several benefits that the Peer Advising Program had on their personal development.

##### Skill building

Peer advisors identified a wide variety of skills obtained from their peer advising experiences with interpersonal communication skills (*n* = 9), organizational and time management skills (*n* = 6), and presentation skills (*n* = 6) most frequently identified. With more experiences, PAs (*n* = 7) gained more confidence in their ability to make presentations and conduct one-to-one advising sessions. The following quotes from PAs’ reflection papers illustrate the variety of skills they obtained from their peer advising experiences.

I have seen myself transform over the course of the year as I learned to take control over my own projects and ideas, feel confident in a role of support for fellow students and be a valuable member of a productive team.

I enjoy asking students about their interests within Public Health, and bouncing ideas off each other about possible career options, course offerings, or extracurricular opportunities. The most rewarding aspect of advising students is when they feel a sense of relief; students leave the office feeling somewhat less stressed and profoundly grateful for my help. When students express their gratitude after brainstorming a solution together, it feels like I made a difference.

Instead of giving a student the answer to his/her question, I try to provide the tools, resources, and information necessary for the student to find the answer. This instills a sense of responsibility and self-efficacy in the student.

I have been told by professors after [a] presentation that I come off to the class [in] a very professional way, I can attribute this to the skill developed as a peer advisor.

##### Feeling valued and supported

Many PAs reported that they felt valued (*n* = 6) and made a difference (*n* = 9) in improving the major and in one-to-one sessions with advisees.

I think the best part about being a peer advisor is that we have the ability to make changes within the major based on students’ interests. We are somewhat like ambassadors for the other public health students. Friends and peers alike come to us with their issues. We analyze them, speak up when necessary, and advocate for change.

Peer advisors feel that they add a valuable insight to being a successful student that a staff or faculty member may not be able to offer.

As undergraduate students, we have a current experience of the public health major that faculty members don’t necessarily have, making us able to better understand the questions and attitudes of students. Some students also find us more approachable as peers their own age and level while they might find an adult to be someone they have to impress or be over-prepared to talk to.

Peer advisors expressed a sense of pride (*n* = 8) when referring to their creation and implementation of departmental projects, representing the department, and identifying strategies to improve major and students’ experience in the major.

Now that I have worked to make the school more functional for the student body, I want to see it succeed even further. I feel an obligation to work to make the major the best it can be.

Two PAs reported students approaching them outside of the Peer Advising Center for academic advice and resources. PAs found their work to be rewarding (*n* = 7). They felt that they made positive changes in the major and ability to provide academic resources and strategies to get involved in public health outside of the classroom (*n* = 8).

##### Engaged in the community

A major goal of the PA program was to emphasis teamwork and build community. Consequently, PAs were required to regularly work together in event planning and conducting workshops. The PAs commented on their increased ability to work well with others (*n* = 7), and their strong sense of community within the major (*n* = 7).

The public health peer advising internship has been one of the most defining and influential experiences of my time here at UMass. The role has made an incredible impact on the UMass community I have worked with and been a part of, and I’m so fortunate to have been given the opportunity to make such a difference and have a strong voice within it.

Without this internship, I would have never found this amazing support system.

##### Gained knowledge of the public health field, major, and University

Peer advisors reported that their PA experiences provided them increased knowledge about academic and professional resources (*n* = 8) that were beneficial to their advisees and themselves. PAs reported an increased understanding of the public health field and careers (*n* = 6), major (*n* = 5), and University.

Now that peer advising has ended and I move on from college to a new chapter in my life, I am going to take everything I have gained through this internship and at UMass with me on my journey. I am going to take my excellent interpersonal, time management, organizational, and networking skills along with so many other acquired skills to my new career in public health. I am leaving this internship with so much more knowledge around public health and how the different domains all come together. I have a much clearer view on what I want out of my career and my life and I am confident I have gained the skills and knowledge necessary to be successful.

Becoming a peer advisor this semester was an amazing experience that enhanced my understanding of the public health major and connected me more with the public health community at UMass Amherst.

##### Cultural competency

Four PAs highlighted their increased knowledge of cultural competency and diversity from the internship. It encouraged them to be non-judgmental and more understanding of other students’ experience. One PA commented on a guest speaker who talked about diversity on campus:

[The guest speaker’s] talk really opened my eyes to all the factors that make students diverse. Race and culture tend to be better-known areas of diversity since they are more apparent, but things regarding sexual orientation and learning disabilities, among others that tend to be forgotten, are equally important when it comes to recognizing diversity among students… This kind of information and perspective was great for becoming a better-rounded peer advisor since we come in contact with so many students and its important to recognize that not everyone is a cookie-cutter student.

#### Perceived challenges of the program

Peer advisors also highlighted several challenges with the program, including: (1) low attendance rates for large advising sessions, (2) difficult advising scenarios, (3) not knowing all of the answers, and (4) variable workload throughout the semester.

##### Low attendance rates for large advising sessions

Since the implementation of the program, PAs have conducted large advising sessions that are tailored to the advising needs of specific class years within the PHS major. For example, at the sophomore advising sessions, PAs share information on study abroad programs, and at the junior advising event, the PAs would talk about volunteer, research, and internship opportunities. Several PAs (*n* = 3) expressed their frustration with the low attendance rates at these advising sessions and possible reasons for low turnout.

Getting a very low attendance at [large advising sessions] demonstrated that this might not be the best approach to getting information to students and also that students prefer to meet with advisors one on one. It was noticed that students are using the advising office more and more.

##### Increase role-play for difficult advising scenarios

Peer advisors (*n* = 5) suggested that they would have benefited from more role-plays especially as it related to dealing with difficult student situations. For example, PAs identified the need for more training related to working with: (1) students who discovered they will not be graduating on time, (2) students who were angry about departmental issues such as a lack of class availability, (3) students who were not motivated, and (4) students who expressed concern over having a lower GPA than required for graduate school.

##### Not knowing all of the answers

Five PAs reported it was difficult when they did not know all of the answers. Several PAs were especially nervous at the beginning of the internship when they were still learning. Further, several of the PAs had not yet taken all of the core public health courses, making it difficult to fully describe each of the courses to advisees. PAs suggested several strategies for this situation, including: (1) referring the student to the Undergraduate Program Advisor or Director, (2) accessing the manual or online material, and (3) telling the student that they would find the answer and email them later in the day.

##### Variable workloads

Four PAs commented on their variable workloads in meeting the departmental and student demands throughout the semester. Departmental events tend to be concentrated during the semester and advising needs are especially high at the beginning of each semester and enrollment periods when there is a large influx of students in the Peer Advising Center. Additionally, many of the PAs are our top students who are often involved in a multitude of extracurricular activities. One PA commented, “*I did learn a great lesson from this semester; do not overcommit yourself*.”

### Survey

Ninety-nine of the 507 majors completed the survey (response rate = 20%). Respondents represented various class years with seven freshman (8%), 29 sophomores (14%), 30 juniors (39%), and 33 seniors (39%). A slightly higher amount of PHS majors on the social science track (59%) responded to the survey compared to those on the science track (41%). Of the respondents, 17% never visited the Peer Advising Center, 49% visited the center once a semester, 29% visited two-to-three times per semester, and 5% visited more than four times per semester. The average rating for their last advising session was 3.8 (*n* = 82) distributed across the following scores 1 (not good) = 1%, 2 = 9%, 3 = 29%, 4 = 34%, and 5 (excellent) = 27%.

## Discussion

The archival records provided data on the undergraduates’ utilization of the Peer Advising Center. The high rate of sophomore (*n* = 195) and juniors (*n* = 130) visiting the center may be related to students wanting information about switching into the PHS major. It is unclear why so few seniors (*n* = 86) accessed the Peer Advising Center. Perhaps, seniors were utilizing other resources on campus that were more relevant to their advising and career needs, such as meeting with Career Services, the Program Advisor and Director, or the Graduate Admissions Staff. Although 83% of the respondents used the advising center one or more times, we do not know how representative that is of all the PHS majors. It is estimated that over 70% of the majors used the Peer Advising Center, but records do not provide the means to determine the exact number. In the future, staff will utilize a different method to better track students who visit the center.

While the program was implemented to assist with the advising load, the evaluation results indicate that there are benefits for both PA and advisees. The PAs gained valuable leadership skills, such as communication, public speaking, organization, and working with others. Although not a main focus for all PAs, learning about cultural competency and applying the skills in practice is important for student success. Thus, we plan to expand our PA training to emphasize meeting the advising needs of students from diverse backgrounds, such as students of color and first generation, international, and LGBTQ students.

All the PAs described having a stronger connection to their major and feeling supported in their role. PAs identified that their ability to participate in leadership roles in the department (i.e., serving on the Undergraduate Faculty Advisory Board, executive committee of PHS Club, and Undergraduate Student Advisory Board) provided them with a voice to make change. Having a stronger connection to the department motivates them to encourage other students to become involved. As they identify department needs, they propose and implement projects to meet these needs.

Helping students select courses and providing students with information about the public health major were two of the top reasons students sought advising services. Consequently, PA training includes much information about the public health field and careers. This training not only benefits the students being advised, but also helps the PAs increase their own career readiness and understanding of career options.

In addition to the required PA reflection paper at the end of each semester, we plan to develop a survey tool that evaluates the specific skills gained and achievement of program objectives to evaluate the impacts of the Peer Advising Program on the PAs. The PAs would complete the survey in the beginning, middle, and end of the internship to identify what skills had been acquired and improved, and to identify additional skills PAs needed to work on. PAs will also receive more one-to-one feedback from both the Head PAs and Program Advisor following each survey.

In general, survey responses across campus continue to be low as students receive surveys from multiple offices and programs on campus. Our response rate of 20% was low, but not atypical for student surveys on campus. In the future, we aim to increase the response rate by offering incentives for survey completion and sending an anonymous electronic survey to students within 30 days after their appointment.

Survey participation across the class years was similar for sophomore, juniors, and seniors with approximately 30% for each class year. In contrast, only 7% of survey participants were freshman. The response rate from freshman was low because most students typically enter the PHS major as sophomores and juniors, so there are relatively few freshmen in the PH major.

Due to students being surveyed at the end of an academic year, recall about their experience may be difficult. To better assess the students’ experiences, we plan to initiate a monthly electronic survey of students who used the center. We believe this will increase respondent rates, and respondents will be better able to assess their experiences because of the shortened duration of time between the advising session and survey. The questions would provide information about what was helpful in the advising session and what needed to be improved. Further, the timely feedback will allow PAs to better identify what they need to work on to improve future advising sessions.

Student satisfaction with advising in general has been an area in need of attention since the number of students began to increase rapidly. The Peer Advising Program was implemented as a way to improve overall satisfaction. The PAs’ unique perspective has been a resource in identifying strategies to improve students’ experience in the PHS major. Student satisfaction with peer advising averaged a 3.8 out of a 5.0 scale. While we hope to see this average improve over the coming years, it is notable that approximately 90% of students who used the Peer Advising Center rated their experience as a 3 (average) or above, and approximately 60% rated it as 4 (very good) or 5 (excellent). More investigation is needed to understand the benefits of peer advising for advisees and factors that make for a positive peer advising interaction for students.

## Conclusion

The Peer Advising Program is a useful program to implement to meet the growing demands of undergraduate public health programs. Results of our work with PAs indicate that PAs gain valuable skills that they can carry into their professional world. The program is also a way to engage students in building community within the major. While this program has many benefits, it also requires intensive training and mentoring by a staff person to prepare students for this role. While these results are preliminary, future program evaluation will more thoroughly assess the perception of student satisfaction with the Peer Advising Program for undergraduate students enrolled in the major. We will also be exploring the role of peer advising in student retention within the major.

## Conflict of Interest Statement

The authors declare that the research was conducted in the absence of any commercial or financial relationships that could be construed as a potential conflict of interest.
